# Ionization and Photofragmentation of Isolated Metalloporphyrin Cations Investigated by VUV Action Spectroscopy**

**DOI:** 10.1002/chem.202101515

**Published:** 2021-07-14

**Authors:** Kaja Schubert, Lucas Schwob, Simon Dörner, Marion Girod, Luke MacAleese, Cornelius L. Pieterse, Thomas Schlathölter, Simone Techert, Sadia Bari

**Affiliations:** ^1^ Deutsches Elektronen-Synchrotron DESY Notkestr. 85 22607 Hamburg Germany; ^2^ Univ Lyon Université Claude Bernard Lyon 1 CNRS UMR 5280 Institut des Sciences Analytiques 5 rue de la Doua 69100 Villeurbanne France; ^3^ Univ Lyon Université Claude Bernard Lyon 1 CNRS UMR 5306 Institut Lumière Matière 69622 Lyon France; ^4^ National Physical Laboratory Hampton Road Teddington TW11 0LW United Kingdom; ^5^ Zernike Institute for Advanced Materials University of Groningen Nijenborgh 4 9747 AG Groningen The Netherlands; ^6^ Institut für Röntgenphysik Georg-August-Universität Göttingen Friedrich-Hund-Platz 1 37077 Göttingen Germany

**Keywords:** dissociation, electronic structure, mass spectrometry, porphyrinoids, VUV action spectroscopy

## Abstract

We investigated the photoionization and fragmentation of isolated metal protoporphyrin IX cations (MPPIX^+^ with M=Fe, Co, Zn) by means of vacuum‐ultraviolet (VUV) action spectroscopy in the energy range of 8.5–35 eV. Experiments were carried out in the gas phase by interfacing an electrospray ionization tandem mass spectrometer with a synchrotron beamline. The mass spectra and partial ion yields show that photoexcitation of the precursor ions predominantly leads to ^.^CH_2_COOH radical side‐chain losses of the macrocycle with additional methyl radical (^.^CH_3_) side‐chain losses. Ionization, in contrast, leads to the formation of the intact ionized precursor and various doubly charged fragments which are mostly due to side‐chain cleavages. Although statistical fragmentation dominates, we found evidence for non‐statistical processes such as new fragments involving for example single and double H_2_O losses, indicating that different relaxation mechanisms are at play upon photoionization compared to photoexcitation. The measured ionization energies were 9.6±0.2 eV, 9.4±0.2 eV and 9.6±0.2 eV for FePPIX^+^, CoPPIX^+^ and ZnPPIX^+^, respectively.

## Graphical abstract

**The isolated metalloporphyrin cations** FePPIX^+^, CoPPIX^+^ and ZnPPIX^+^ have been studied in the gas phase by action spectroscopy in the vacuum‐ultraviolet (VUV) range using electrospray ionization tandem mass spectrometry and synchrotron radiation. The ionization energy of each compound has been measured. Photoexcitation and photoionization lead to strikingly different fragmentation pathways. Evidence for non‐statistical dissociation processes including water losses were identified. Fragment assignments have been supported by additional high‐resolution collision‐induced dissociation experiments on a hybrid quadrupole‐orbital mass spectrometer.

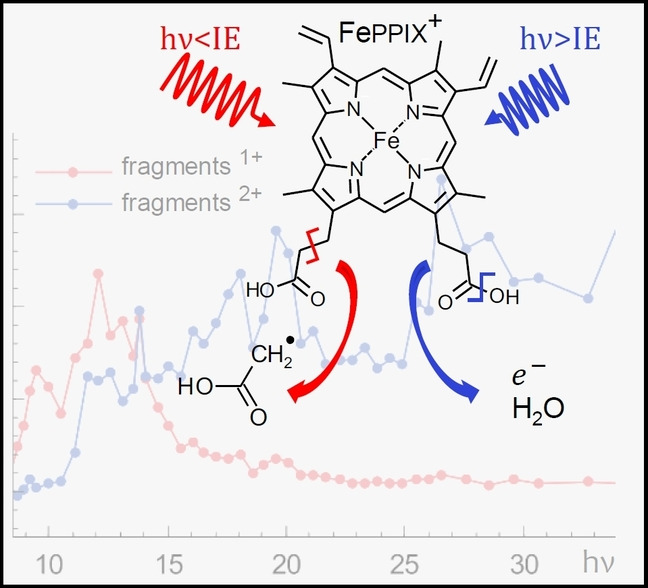

## Introduction

**Figure 1:**
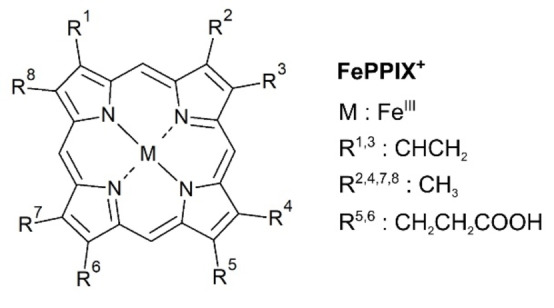
Structure of the iron protoporphyrin IX cation (FePPIX^+^).

Metalloporphyrins are molecules widely found in nature. They are for instance involved in photosynthesis and in oxygen transport by the red blood cells of vertebrates.[^1^, ^2^] Moreover, metalloporphyrins find applications in many other fields such as solar cells,^[3]^ photodynamic therapy[^4^, ^5^] or radiotherapy.[^6^, ^7^, ^8^] Metalloporphyrins are highly correlated systems consisting of a tetrapyrrole ring with a metal ion in its center. In nature, several peripheral side chains are attached to the porphyrin ring, as shown in Figure 1 for the cofactor of hemoglobin, namely the iron protoporphyrin IX (FePPIX), better known as the heme group.

Metalloporphyrins are extensively studied in the solid and in the liquid phase (see for example[^9^, ^10^] and references therein). However, agglomeration of the metalloporphyrins and the binding of axial ligands to the metal center can affect the properties of the metalloporphyrins. Performing studies in the gas phase, in contrast, allows to rule out the influence of the solvent and to focus on the intrinsic properties of the metalloporphyrins. Previous experimental gas‐phase studies have focused on free‐base porphyrins and on metal tetraphenyl or metal octaethyl porphyrins which can be transferred to the gas phase by for example sublimation.[^11^, ^12^, ^13^, ^14^] Yet, this method is usually not applicable for naturally occurring protoporphyrins due to their high fragility. It has been shown that they may be transferred to the gas phase via laser vaporization.^[15]^ Another option is to exploit soft‐ionization techniques such as electrospray ionization which, in addition, allows the application of mass selection and ion trapping techniques. Previous studies on electrosprayed protoporphyrins have focused on collisional activation,[^16^, ^17^, ^18^] electron‐capture dissociation^[19]^ and on photoexcitation experiments in the optical regime at the Soret band (380–450 nm) and Q bands (500–600 nm).[^16^, ^20^, ^21^, ^22^, ^23^, ^24^, ^25^, ^26^, ^27^, ^28^] Photoexcitation experiments of metal protoporphyrin cations at the Soret band and Q bands have shown that the two competing relaxation pathways in the optical regime are 1. direct internal conversion leading to peripheral side‐chain cleavages or 2. intersystem crossings, internal conversion and subsequent side‐chain losses.[^23^, ^24^, ^25^] The lowest energetic fragmentation channels reported are the *β*‐cleavages leading to radical single and double ^.^CH_2_COOH side‐chain losses.[^16^, ^20^, ^24^] Recently, we have studied the electronic structure of isolated CoPPIX^+^ and its photofragmentation after cobalt inner‐shell excitation in the soft X‐ray regime.^[29]^ The post‐ionization side‐chain cleavages observed were differing, to some extent, from optical excitation experiments, suggesting that different mechanisms are involved in the formation of these fragments. Furthermore, we observed that a specific deexcitation mechanism is involved upon inner‐shell excitation of the metal center and subsequent ionization leading to for example water losses from the photoionized precursor. The question was raised whether this mechanism is related to the absorption site, namely the core level of the metal center, or to the ionization process in general.

Using photons in the vacuum‐ultraviolet (VUV) energy range as a probe allows to examine processes induced by photoexcitation and ionization in the valence levels of metalloporphyrins. Understanding how metalloporphyrins react towards ionizing radiation is important, for example, for radiotherapy applications as the metal atom embedded in the metalloporphyrins can act as an antenna for the radiation and as an intense source of secondary low‐energy electrons and reactive oxygen species, both known to induce substantial damage in cells.[^7^, ^8^, ^30^, ^31^, ^32^] Moreover, the ionization energy is an important parameter to characterize the reactivity of molecules. From theoretical studies it is known that ionization of metalloporphyrins induces ultrafast charge redistribution processes,^[33]^ which can determine the subsequent relaxation pathways of the molecules. In principle, such processes can be probed in time‐resolved pump‐probe experiments. Especially, with the development of high harmonic generation (HHG) light sources, time‐resolved photofragmentation experiments with 15 to 35 eV photons became possible in the laboratory. Knowledge of the photofragmentation products in this energy region is an important first step in the design of such experiments.

VUV action spectroscopy has shown its value in previous photofragmentation studies on isolated peptides, by coupling electrospray ionization tandem mass spectrometers (ESI‐MS) to synchrotron beamlines. Such experiments have been pioneered a decade ago, providing information on deexcitation mechanisms after photoexcitation and ionization.[^34^, ^35^, ^36^, ^37^, ^38^, ^39^] As metalloporphyrins cations can be transferred into the gas phase with electrospray ionization sources, in this study, we could apply for the first time VUV action spectroscopy to isolated metal protoporphyrin IX cations (FePPIX^+^, CoPPIX^+^, ZnPPIX^+^) by interfacing an ESI‐MS instrument to the U125‐2_NIM VUV beamline of the BESSY II synchrotron (Helmholtz‐Zentrum Berlin). The experiments have been carried out in the energy range of 8.5–35 eV to investigate both, photoionization and photodissociation processes. In the following, we first focus on FePPIX^+^ and further characterize the influence of the metal type on the ionization energy and deexcitation pathways by a comparison with cobalt and zinc protoporphyrin IX cations. In order to help assigning isobaric fragments, collision‐induced dissociation (CID) data have been recorded for FePPIX^+^ on a high‐resolution tandem mass‐spectrometer.

## Results and Discussion

### VUV photoabsorption of FePPIX^+^

**Figure 2:**
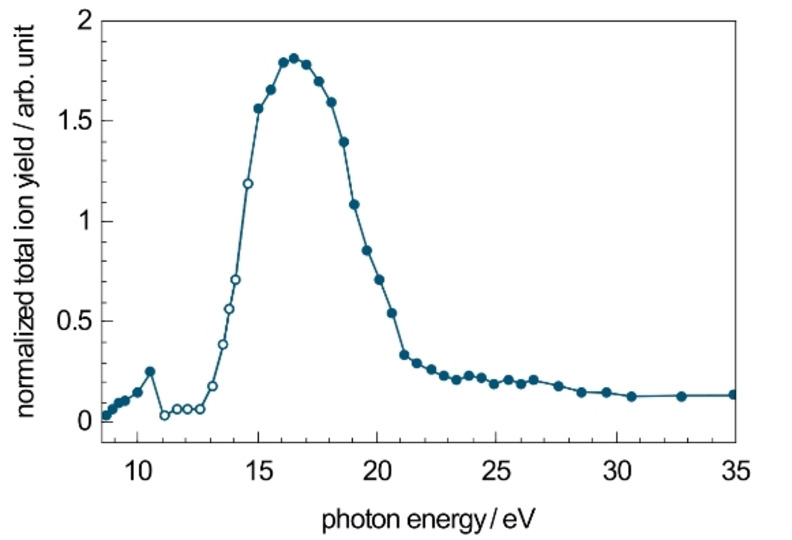
Total ion yield (TIY) of FePPIX^+^ after irradiation with VUV photons at 8.5–35 eV normalized to the number of photons and the precursor ion yield. At photon energies between 11 and 15 eV (open circles) high harmonic photons contribute to the measured photon flux.

The total ion yield (TIY) of FePPIX^+^ after irradiation with VUV photons normalized to the number of photons and precursor ions is shown in Figure 2. The TIY is obtained here by summing up the yield of the main cationic photo products, excluding the precursors ions. The normalized TIY reflects the absorption cross section of FePPIX^+^ and follows overall a Gaussian trend with a maximum absorption cross section at ∼16.6 eV. Note that at photon energies between 11 and 15 eV high harmonic photons from the undulator contribute to the measured photon flux leading to an overestimation of the number of photons and accordingly an underestimation of the absorption cross section in Figure 2.

### Characterization of the photo products

**Figure 3:**
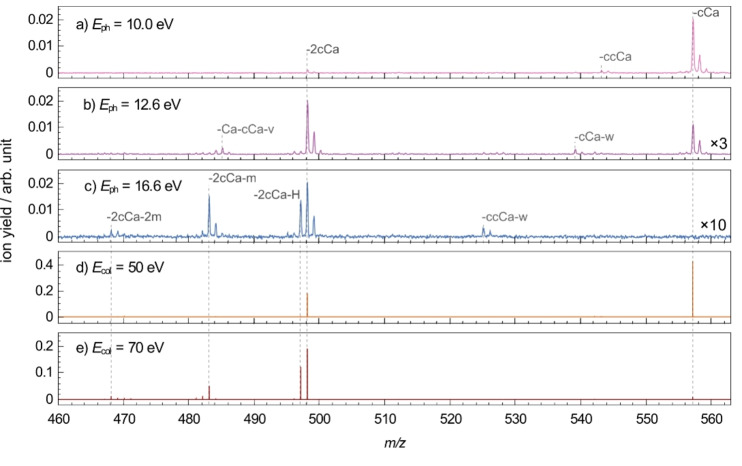
Mass spectra of FePPIX^+^ in the singly charged *m/z* region after irradiation with VUV photons at *E*_ph_ a) 10.0 eV b) 12.6 eV (upscaled by factor 3) c) 16.6 eV (upscaled by factor 10), and after collisional activation at collision energies (*E*_col_) of d) 50 eV and e) 70 eV. The mass spectra are normalized to the respective TIY. The peak assignments are indicated. Note that in d) and e) the monoisotopic precursor ion was selected, whereas in a), b) and c) all isotopes were included.

**Figure 4:**
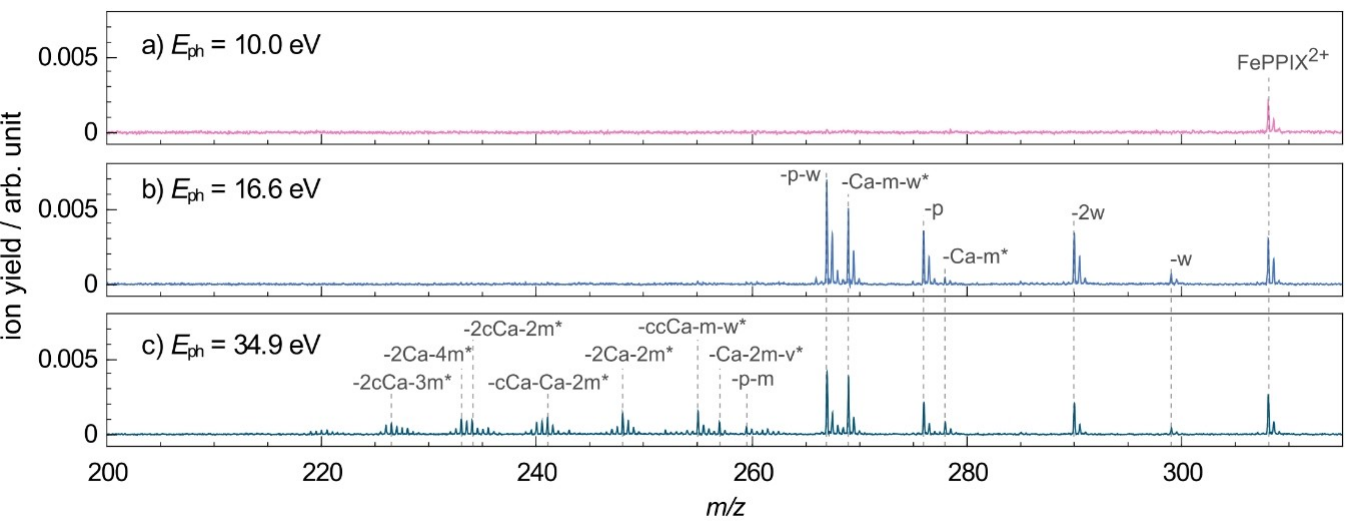
Mass spectra of FePPIX^+^ in the doubly charged *m/z* region after irradiation with VUV photons at a) 10.0 eV, b) 16.6 eV and c) 34.9 eV. The mass spectra are normalized to the respective TIY. The peak assignments are indicated. Peaks labelled with * have multiple possible assignments which are listed in Table 1.

After irradiation with VUV photons, both singly charged fragments at 465<*m/z*<565 and doubly charged photo products at 200<*m/z*<320 are formed. Full range mass spectra are shown in Figure S1. Here, spectra in the mass ranges of the singly (Figure 3) and doubly (Figure 4) charged photo products are presented and discussed separately. The yields of singly and doubly charged photo products depend on the excitation energy, as can be seen in the mass spectra in Figure 3 and Figure 4. Their excitation energy dependence will be discussed in more detail in the next sections of the discussion.

The mass spectra of singly charged fragments formed after photoabsorption of VUV photons at 10.0 eV, 12.6 eV and 16.6 eV are shown in Figure 3a–c, respectively. The three photon energies represent different photoexcitation regimes that lead to the formation of singly charged fragments. The observed photo products can be formed by different combinations of bond cleavages leading to peaks with similar *m/z*. For unambiguous peak assignments, CID experiments with a high‐resolution hybrid quadrupole‐orbitrap setup were performed. Mass spectra after collisional activation at 50 eV and 70 eV are shown in Figure 3d–e, respectively. At a collision energy of 50 eV, the main peaks at *m/z* 557.1571 and 498.1446 are due to *β*‐cleavages on the carboxylic side chains and are respectively assigned to the radical losses of one (−59 u, −CH_2_COOH, −cCa) and two (−118 u, −2cCa) acetic acid groups from the porphyrin ring (Figure 3d). These fragmentation channels are known as the lowest energetic fragmentation channels of FePPIX^+^ from previous CID and laser‐induced dissociation studies.[^16^, ^40^] At a collision energy of 70 eV, the −cCa peak is absent, whereas the −2cCa loss is accompanied by an additional hydrogen loss, −2cCa−H, at *m/z* 497.1377 (Figure 3e). It is noteworthy that, rather than an independent −H loss, a hydrogen rearrangement could lead to a neutral loss of CH_3_COOH (−60 u), which in addition to a radical −cCa loss would yield a peak at the same *m/z*. However, the single loss of CH_3_COOH, which could be thus expected, is not observed. Although the present data do not allow a definite conclusion, we will use the −2cCa−H assignment in the following.

Peaks at *m/z* 483.1219 and 468.0985 arise from additional sequential radical methyl (−15 u, −^.^CH_3_, −m) losses besides the −2cCa loss: −133 u, −2cCa−m and −148 u, −2cCa−2m, respectively. This is in accordance with a collisional‐activation study reporting on sequential methyl losses subsequent to the −cCa loss.^[16]^ Other fragment peaks can be attributed to different combinations of −cCa, −m, −H, radical propionic acid side‐chain losses (−73 u, −^.^CH_2_CH_2_COOH, −ccCa) and vinyl losses (−27 u, −CHCH_2_, −v). A more detailed list of the observed fragments can be found in the Supporting Information Table S1. All assignments were supported by exact masses with errors <±15 ppm. Note that no CID fragment was observed below *m/z* 465 (see Figure S1).

In the VUV case, in Figure 3a–c, fragment peaks appear at the same *m/z* as after CID in the singly charged *m/z* region and are assumed to arise from the same neutral radical side‐chain losses from the porphyrin ring, as discussed in more detail in the next parts.

The mass spectra of doubly charged photo products formed after photoabsorption of VUV photons at 10.0 eV, 16.6 eV and 34.9 eV are shown in Figure 4, respectively representing photoabsorption regimes at the onset of the ionization threshold, at the maximum of the TIY spectrum and far above the absorption band. The peak at *m/z* 308.1 corresponds to the intact ionized precursor ion, namely the FePPIX^2+^ dication. The fragment peaks at *m/z* 299.1 and 290.0 can be assigned to the single (−18 u, −H_2_O, −w) and double (−32 u, −2w) water loss, respectively. Note that water losses contribute comparatively weakly to the VUV photofragmentation in the singly charged region (Figure 3). However, we have observed such loss channels after soft X‐ray excitation of CoPPIX^+^.^[29]^

Other fragment peaks in Figure 4a–c arise from various multiple cleavages of the peripheral side chains of the porphyrin ring, including water loss (Table 1). Remarkably, the main singly charged fragments formed after VUV photoabsorption or collisional activation, namely single and double −cCa loss, do not appear as losses from the doubly charged species in Figure 4a–d. The minor peaks at *m/z* 278.5 (−cCa) and 249.0 (−2cCa) are assigned to isotopes of the *m/z* 278.0 and 248.5 fragments, respectively.

**Table 1:** Assignments and appearance energies *E*_a_ of doubly charged photo products formed after VUV photoionization of FePPIX^+^. With photon energy steps of 0.5 eV and an energy bandwidth of less than 0.1 eV, the uncertainty of the *E*_a_ is given as (*E*_a_±0.3) eV. Photo products for which the PIY is shown in Figure 6 are printed in bold. Other PIYs can be found in the Supporting Information Figure S4.

m/z	Assignment	E_a_/eV	m/z	Assignment	E_a_/eV
308.1	**FePPIX^2+^**	9.75	241.5	−2cCa−m/−ccCa−Ca−m/−ccCa−m−w−v/−4m−ccCa	20.5
299.1	−w	12.0	241.0	−cCa−Ca−2m/−cCa−2m−w−v	20.0
290.0	**−2w**	12.5	240.5	**−2Ca−3m/−Ca−3m−w−v/−3m−2w−2v**	22.0
278.0	−Ca−m/−m−w−v/−4m	14.5	240.0	−ccCa−3m−w/−p−3m−v	21.5
276.0	**−p**	14.0	235.5	−2cCa−v/−ccCa−Ca−v/−ccCa−w−2v/−3m−ccCa−v	21.0
269.0	−Ca−m−w/−m−2w−v/−4m−w	13.5	234.0	−2cCa−2m/−ccCa−Ca−2m/−ccCa−2m−w−v	24.0
267.0	**−p−w**	13.5	233.5	−cCa−Ca−3m/−cCa−3m−w−v	23.0
259.5	**−p−m**	15.5	233.0	**−2Ca−4m/−Ca−4m−w−v/−4m−2w−2v/−cCa−p−v**	24.5
256.0	−cCa−Ca/−cCa−w−v/−cCa−3m	–	227.5	−2ccCa−m/−cCa−Ca−2m−v/−cCa−2m−w−2v	24.5
255.0	−ccCa−m−w/−p−m−v	15.5	227.0	−cCa−ccCa−2−m/−2Ca−3m−v/−Ca−3m−w−2v	25.0
248.0	**−2Ca−2m/−Ca−2m−w−v/−2m−2w−2v**	17.5	226.5	−2cCa−3m/−ccCa−Ca−3m/−ccCa−3m−w−v	27.0
247.5	−ccCa−2m−w/−p−2m−v	19.0	226.0	−cCa−Ca−4m/−cCa−4m−w−v/−2p−2w/−ccCa−p−v	26.5

In addition, we observe peaks at *m/z* 276.0 and 267.0, corresponding to losses of −64 u and −82 u, which are not present as singly charged species and do not match any combination of side‐chain cleavages. In our previous study upon metal inner‐shell excitation of CoPPIX^+^ we assigned the −64 u and −82 u losses to the loss of a pentadiyne (−C_5_H_4_, −p) fragment and an additional water molecule (−p−w), respectively.^[29]^

### Energetics of the collisional activation of FePPIX^+^

Collisional activation of FePPIX^+^ leads to statistical fragmentation subsequent to the intramolecular vibrational redistribution of the internal energy. Therefore, one would in general expect an increasing number of cleavages with increasing collision energies, which is consistent with the fragmentation at collision energies of 50 and 70 eV (Figure 3d and Figure 3e, respectively).

For a more detailed analysis, the energy of the center of mass *E*_com_ is considered which is the maximum energy which can be converted into internal energy via a single collision event. It is given by $E_{com}=\;E_{lab}\;\cdot \;m_{gas}/\left(m_{gas}+m_{ion}\right)$ , where *m*_gas_ is the mass of the collision gas, *m*_ion_ the mass of the precursor ions and *E*_lab_ the collision energy in the laboratory frame. In a single collision event at collision energies of 50 eV maximum 2.2 eV can be converted into internal energy, for 70 eV *E*_com_=3.0 eV. In addition, multiple‐collision events can contribute to the fragmentation. The probability of multiple‐collision events increases with increasing collision energies.

In a previous CID and theoretical study of FePPIX^+^, the dissociation energy for the first acetic acid side chain is reported to be *D*_1_(cCa)=1.9±0.2 eV.^[16]^ In line, the −cCa side‐chain fragment is the main fragment for *E*_com_=2.2 eV (Figure 3d). The ‐2cCa loss in Figure 3d, with *D*_2_(cCa)=2.4±0.3 eV, is formed by at least two collisions. Dissociation energies for subsequent other side‐chain losses are reported to follow *D*(cCa)<*D*(methyl)<*D*(vinyl). Thus, we assume that more collisions are required to form fragments involving −2cCa and methyl losses. Considering multiple‐collision events, our results are in accordance with the study of Charkin et al.^[16]^

### VUV photofragmentation of FePPIX^+^

**Figure 5:**
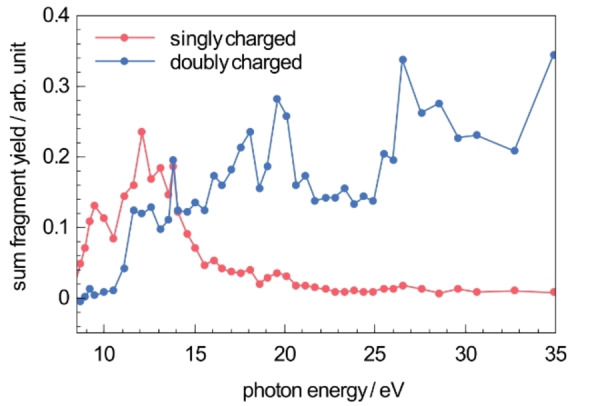
Summed ion yields of all singly charged and doubly charged photo products after VUV photoabsorption of FePPIX^+^. The ion yields are normalized to the TIY.

Singly and doubly charged photo products are both formed after photoabsorption at the same photon energy, as can be seen for example in the mass spectra of Figure 3c and Figure 4b obtained at 16.6 eV. This is furthermore illustrated in Figure 5 which presents, as a function of the photon energy, the branching ratios for the production of singly charged and doubly charged photo products following photoexcitation and photoionization, respectively. These partial ion yield (PIY) spectra provide additional information on the appearance energies *E*_a_ of the photo products, as well as on the branching ratio of photo products formed after photoabsorption at a specific excitation energy.

**Figure 6:**
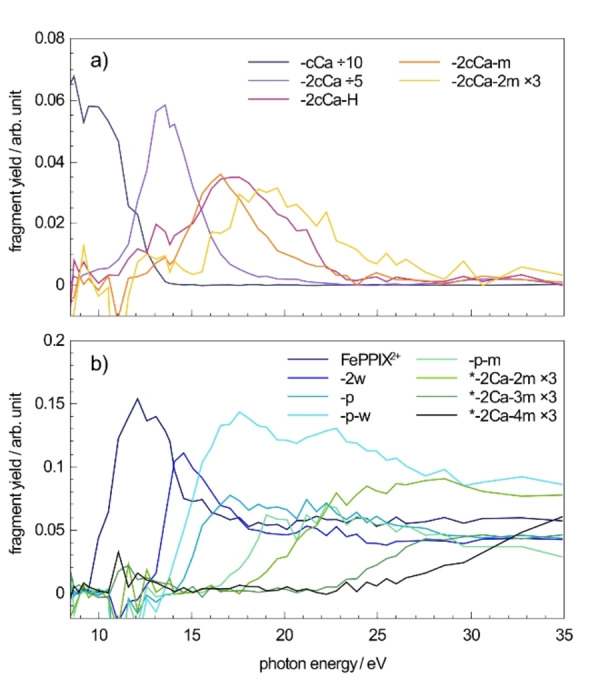
Partial ion yield (PIY) spectra for a) singly charged and b) doubly charged photo products after VUV photoabsorption of FePPIX^+^. The PIYs are normalized to the TIY. For better comparison between the ion yields and reading of the appearance energies, the PIY of −cCa and −2cCa are divided by a factor 10 and 5 respectively, while −2cCa−2m and *−2Ca−*x*m (*x*=2, 3, 4) are multiplied by a factor 3.

The PIY spectra for the main photo products are shown in Figure 6. PIY of other photo products can be found in the Supporting Information Figure S4. Moreover, the PIY spectrum of the ionized precursor allows an estimate of the ionization energy of the system. Here, the ionized precursor appears at excitation energies above 9.5 eV. With a photon energy step size of 0.25 eV around the ionization energy and a photon energy bandwidth less than 0.1 eV, the ionization energy of FePPIX^+^ is determined to be 9.6±0.2 eV. For a more accurate determination of the ionization energy, more datapoints are needed below the appearance energy of the dication. For neutral metalloporphyrins the ionization energy $E_{I}^{0}$ is in the range of 6–6.5 eV.[^11^, ^12^, ^41^] The ionization energy for the cation (*z*=1) can be approximated by adding an extra term which considers the initial charge on the molecule, as previously applied in Ref. [42] for proteins:

$$E_{I}\left(z\right)=E_{I}^{0}+e^{2}\;\cdot \;z\;\cdot \;\frac{1}{4\pi \epsilon R_{m}\left(z\right)}$$

where *R_m_* (*z*) is the radius of the porphyrin ring, *ϵ* is the absolute permittivity of the medium and *e* the electrical charge. *R_m_* (*z*) is here the maximum distance of the metal center and a carbon atom of the porphyrin ring (*R_m_* (*z*)=4.27 Å) and is taken from the DFT‐optimized geometry of Co^III^PPIX^+^.^[29]^ If an ionization energy for neutral FePPIX of 6.25 eV is assumed, the calculated ionization energy of 9.6 eV is within the uncertainty window of the experimentally determined value.

Below the ionization threshold some singly charged photo products for example [FePPIX−cCa]^+^ are observed showing that these photo products are formed by photoexcitation and subsequent neutral losses from the precursor ion. We assume that other singly charged photo products are mainly formed after photoexcitation, too. Indeed, even above the ionization threshold, besides photoionization, photoexcitation from deep valence orbitals contributes to the photoabsorption (see for example Ref. [43,44]). The contribution from singly charged products from photoexcitation is expected to decrease with increasing photon energy, which is confirmed in Figure 5.

The PIY spectra of the singly charged fragments in the VUV energy region are dominated by the single −cCa loss and the double −cCa loss with *E*_a_=10.5±0.3 eV. Sequential single and double methyl losses accompanying the −2cCa loss appear at increasing energies of ∼13 eV and ∼15 eV, respectively, supporting the conclusion that internal conversion, intramolecular vibrational energy redistribution and statistical fragmentation are involved after VUV photoexcitation. It is noteworthy that the experimental values show a discrepancy with regards to the reported calculated dissociation energies of the methyl losses following the −2cCa loss: *D*(m)=4.2–4.6 eV.^[45]^ The additional hydrogen loss besides −2cCa appears at slightly higher energies as the −2cCa fragment. The PIY extends over a relatively wide range (compared to for example the −2cCa−m fragment in Figure 6), which can be due to the hydrogen loss from different sites of the molecule with slightly different dissociation energies.

Above the ionization threshold the doubly charged fragments are mainly formed by neutral losses from the ionized precursor. It is worth noting that neither the intact triply ionized precursor nor any triply charged fragments are observed in the mass spectra. Metalloporphyrins are highly correlated systems and processes such as electron shake‐offs play a role already at a few eV above the ionization edge.^[33]^ Therefore, we would expect double electron removal to be involved at higher excitation energies after VUV photoabsorption. On the one hand, the absence of triply charged photo products suggests that double electron removal does not happen. On the other hand, double electron removal could lead to charged side‐chain losses, where fragments would contribute to the observed doubly charged fragments. The charged side chains cannot be detected in the present experiment due to the low‐mass cut‐off of the spectrometer and thus, charged side‐chain losses cannot be strictly excluded. Still, we assume that single ionization dominates in this energy region and that doubly charged fragments are predominantly formed by neutral losses from the dication FePPIX^2+^. The fragmentation pathways after photoexcitation and photoionization differ in several aspects.

After photoionization, the water loss is more strongly involved in the fragmentation and new types of photofragments are formed such as the −p and −p−w fragments. Furthermore, the major losses observed from the photoexcited precursor are essentially absent from the photoionized precursor. This indicates that different deexcitation mechanisms are involved in the formation of the singly and doubly charged photo products. Notably, the fragmentation after VUV photoionization of FePPIX^+^ at higher *m/z*’s (265<*m/z*<320) strongly resembles the fragmentation after irradiation of CoPPIX^+^ with soft X‐rays at the metal L‐edge,^[29]^ suggesting that the underlying dissociation mechanisms are related to the ionizing radiation and not specifically to the absorption site. At *m/z*<265 less cleavages are observed after irradiation with VUV photons compared to the soft X‐rays which can be due to a difference in internal energy conversion. Another possibility would be that higher charge states, which can be reached after cobalt inner‐shell excitation and subsequent Auger cascade decays, may lead to a stronger Coulomb repulsion and thus to the formation of these fragments in the X‐ray case.

Appearance energies and peak assignments for the different photo products are grouped in Table 1. The general trend is that the higher the excitation energy, the more cleavages are involved in the fragmentation. This indicates that internal conversion, intramolecular vibrational redistribution and statistical fragmentation in the hot ground state of the dication are involved in the photoionization case after VUV photoabsorption.

The two fragments with appearance energies closest to the ionization threshold are the −H_2_O and −2H_2_O fragments, showing that the water loss plays a special role and is facilitated in the metalloporphyrin dication formed after photoionization. As was observed in protonated free‐base porphyrins^[17]^ and fatty acids (see for example Ref. [46] and references therein), the water losses are likely to involve a proton transfer from one carboxylic group to the other and to happen in the protonated COOH_2_^+^.

Interestingly, the −p and −p−w fragments are exceptions to the general trend as the −p−w fragment appears at lower excitation energies than the −p fragment (Figure 6, Table 1). Note that a similar behavior is observed for the −m−Ca and −m−Ca−w fragments (Table 1 and Figure S4). One explanation may be that the energetics of −p or −m−Ca losses become more favorable once a water is already lost from the dication. In the case of truly independent losses involving statistical fragmentation, one would expect a higher appearance energy for the water‐loss involving fragments. This seems to be the case, for instance, for the −p−m fragment which is formed at higher energies than the −p fragment as one would expect in terms of internal energy. When statistical fragmentation is not in play, it would imply that different, localized, dissociation processes are favoring the water loss following photoabsorption at 12–14 eV. The determination of the exact mechanisms leading to these losses is, though, beyond the scope of this paper. MS^*n*^ or time‐resolved experiments would be necessary to assess the exact process involved here.

### The influence of the metal type

**Figure 7:**
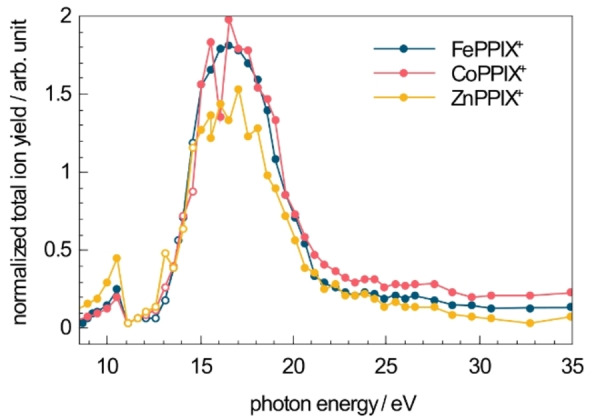
Total ion yield (TIY) for FePPIX^+^, CoPPIX^+^ and ZnPPIX^+^ after irradiation with VUV photons at 8.5–35 eV normalized to the number of photons and the precursor ion yield. At photon energies between 11 and 15 eV (open circles) high harmonic photons contribute to the measured photon flux.

To investigate the influence of the metal ion on the ionization and fragmentation of metalloporphyrins, similar experiments were carried out for CoPPIX^+^ and ZnPPIX^+^. The normalized TIY for CoPPIX^+^ and ZnPPIX^+^ after VUV photoabsorption are shown in Figure 7, representing their respective absorption spectra. For comparison, the absorption spectrum of FePPIX^+^ from Figure 2 is reproduced. The absorption spectra for all metal ions essentially overlap, with a maximum at ∼16.6 eV. This suggests that the metal type has a minor influence on the absorption properties in the present energy region. Despite this similar trend, it appears that ZnPPIX^+^ exhibits a lower, by up to 25 %, absorption cross section than the two other protoporphyrins in the VUV range. Similarly, ionization energies are determined from the appearance of dications at 9.4±0.2 eV for CoPPIX^+^ and 9.6±0.2 eV for ZnPPIX^+^, only slightly deviating for the different metals.

**Figure 8:**
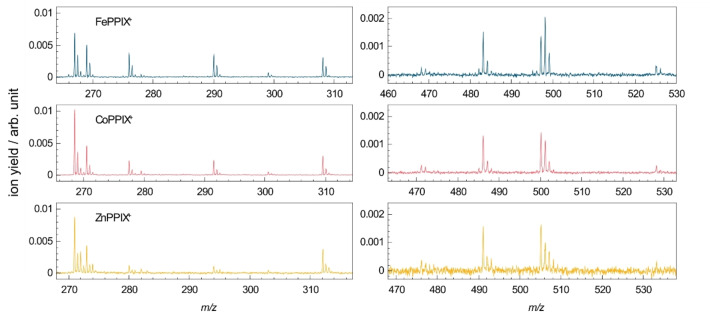
Mass spectra for FePPIX^+^, CoPPIX^+^ and ZnPPIX^+^ after irradiation with 16.6 eV photons for the singly charged (right) and doubly charged (left) photo products. The *m/z* axes are shifted by the difference in mass between Co and Fe (Δ*m*=3.0 u) and Zn and Fe (Δ*m*=8.0 u), respectively.

Figure 8 compares the photofragmentation mass spectra of the three metalloporphyrins at 16.6 eV. The fragment‐rich mass spectra at 34.9 eV are shown with an extended *m/z* range for the doubly charged fragments region in the Supporting Information Figure S6. The *m/z* axis is plotted such that the different masses of the metal ions are considered and photo products can be directly compared. Note that the isotopic distributions for the metals are different as shown in Figure S5 for the precursor ion peaks. The isotopic distribution for FePPIX^+^ and CoPPIX^+^ are similar, whereas the zinc isotopes lead to a broader *m/z* distribution. Overall, the photo products formed after VUV photoabsorption of the different metal protoporphyrins (FePPIX^+^, CoPPIX^+^ and ZnPPIX^+^) are similar and only differ by the *m/z* of the metal ion. In addition, the photo products follow similar trends in their PIY as shown in the Supporting Information (Figures S3 and S4). Therefore, we conclude that, despite the changes in the electronic structure due to the different metal ions, the fragmentation pathways of the metal protoporphyrins are not influenced by the metal type.

## Conclusion

The ionization and fragmentation of isolated cationic metal protoporphyrins (FePPIX^+^, CoPPIX^+^ and ZnPPIX^+^) after VUV photoabsorption was studied by VUV action spectroscopy in the energy range of 8.5–35 eV. The ionization energies were determined with values of 9.6±0.2 eV, 9.4±0.2 eV and 9.6±0.2 eV for FePPIX^+^, CoPPIX^+^ and ZnPPIX^+^, respectively. In general, the absorption and fragmentation of the different metals were similar, with a maximum absorption at around 16.6 eV. The collision‐induced dissociation spectra, obtained at high mass resolution, allowed us to lift some past ambiguity regarding the attribution of isobaric fragments. After photoexcitation, the fragmentation is governed by *β* ‐cleavages of the carboxylic acid side chain and followed by methyl losses, similarly to the collision‐induced dissociation case. This observation suggests that fast internal conversion followed by intramolecular vibrational energy redistribution is involved after VUV photoexcitation and leads to a statistical fragmentation similar to the CID case. However, photoionization leads to the formation of new, exclusively doubly charged, fragments, indicating that different deexcitation mechanisms are involved after ionization. This includes single and double water loss from the ionized precursor. The partial ion yields of, for example, −p−w fragments suggests that the water loss is a facile process. The corresponding fragments without the accompanying water loss are only formed at higher photon energies. Investigating the exact mechanisms involved for these water losses will be the focus of future work. In addition, similar to the photoexcitation regime, more cleavages were observed with increasing photon energy, suggesting that after ionization, the system quickly relaxes to the hot ground state of the dication and undergo, essentially, statistical fragmentation.

## Experimental Section

### Sample preparation

For both, VUV photoabsorption and collisional‐activation experiments, the samples were prepared in the same way. Zinc (II) Protoporphyrin IX (≥95 %) was purchased from Avantor and Hemin (≥90 %), Protoporphyrin IX cobalt chloride (100 %), methanol, dichloromethane and formic acid were purchased from Merck. All chemicals were used without further purification. The sample solutions were prepared at 30 μM concentration in methanol and dichloromethane (1 : 1 in volume) with 1vol% formic acid.

### VUV photoabsorption experiments

In the present study, a home‐built tandem mass spectrometer, described in more detail in Ref. [47], was interfaced with the U125‐2_NIM beamline[^48^, ^49^] of the BESSY II synchrotron (Helmholtz‐Zentrum Berlin, Germany). The metalloporphyrin cations were formed by electrospray ionization (ESI) of the sample solution. The ions were focused in an ion funnel, guided through a radiofrequency octupole and filtered by their mass‐to‐charge *m/z* ratio with a quadrupole mass filter. The precursor ions were then accumulated and kinetically cooled in a 3D Paul trap filled with helium buffer gas before being irradiated with VUV photons. The low‐mass cut‐off of the ion trap was at *m/z*≈80. Cationic photo products and precursor ions were extracted into a reflectron time‐of‐flight mass spectrometer (*m*/Δ*m*≈2400) and detected with a microchannel plate detector (MCP). The mass calibration is based on the well‐known photofragmentation mass spectrum of leucine enkephalin,[^34^, ^50^] which was obtained under similar conditions as the photofragmentation spectra of the metalloporphyrins. Our mass spectra confirmed that the precursor ions were in the cationic [M^III^PPIX]^+^ form and not in the protonated [M^II^PPIX+H]^+^ form (Figure S2).

The energy of the photons delivered by the synchrotron beamline was scanned across the range of 8.5 to 35 eV by steps of 500 meV. From 8.5 to 10 eV, the energy steps were reduced to 250 meV in order to measure the ionization energy with better precision. The photon energy bandwidth was kept below 100 meV by adjusting the beamline exit slit accordingly. For photon energies above 25.5 eV the beamline slits were opened to 300 μm and up to 1800 μm for photon energies below 14.5 eV. Moreover, to filter out contributions from high harmonic photons which significantly contribute to the photon beam at energies below 15 eV, a MgF_2_ filter was moved into the photon beam path upstream of the ion trap. Due to the strongly decreasing transmission at photon energies above 11 eV, the MgF_2_ filter could be only used at photon energies below 11 eV. The photon flux was measured with a calibrated photo diode downstream of the ion trap. The irradiation time of the trapped precursor ions, typically ranging from 0.15 to 9.7 s, was controlled with a mechanical beam shutter and was set such that the relative depletion of the precursor ions was ∼10 %. In consequence, the contribution of photo products formed by sequential absorption of two photons is <10 % and considered negligible when discussing our results.

To account for the long time‐scale fluctuations of the electrospray ion source, single acquisitions with (precursor+photons) and without (precursor‐only) irradiation of the precursor ions were subsequently recorded and repeated in a duty cycle mode. Typically, 150 precursor+photons and 75 precursor‐only spectra were acquired and averaged, separately, for each photon energy step. At each photon energy, the background in the precursor‐only spectrum (from collisional activation of the precursor ions in the buffer gas) was subtracted from the associated precursor+photons spectrum. Contributions of photofragments from the residual gas to the background were only observed at *m/z*<150 in photons‐only spectra. Since we focus on the *m/z*>200 region in our discussion, subtraction of the photons‐only background was not applied.

Partial ion yields (PIYs) were obtained for the main cationic photo products of the metalloporphyrins by summing up the yield of their corresponding peak in the background‐corrected mass spectrum at each energy step. In the absence of the MgF_2_ filter at photon energies above 10.5 eV the high harmonic photons contribute to the measured photon flux. A correction factor was calculated by using the yield of a photofragment which is exclusively formed by absorption of higher harmonic photons (e.g. *m/z* 255). The procedure is described in more detail in the Supporting Information. The correction factors were applied to the respective PIYs. Moreover, all PIYs were corrected by the detection efficiency of the MCP, *P*, of products with different *m/z*‘s based on the semiempirical equation established earlier:^[51]^

$$P=\frac{1}{2}\;\left(1+\tanh\left(\frac{v-28500}{11000}\right)\right)$$

where *v* is the impact velocity of the detected particle in m s^−1^.

The total ion yield (TIY) is given by summation of all PIYs at each photon energy. All shown mass spectra and PIY spectra are normalized to the respective TIY. This way of normalization gives an impression about the branching ratio of photo products at one photon energy.

### Collision‐induced dissociation experiments

For the CID experiments a hybrid quadrupole‐orbitrap Q‐Exactive® mass spectrometer (Thermo Fisher Scientific, San Jose, CA, USA) equipped with a heated ESI source was used. FePPIX^+^ cations were formed by electrospray ionization of the sample solution and infused at a flow rate of 5 μL/min. The ESI source was operated in positive ion mode with a spray voltage of 4500 V. The sheath gas and auxiliary gas (nitrogen) flow rates were set at 20 and 15 (arbitrary unit), respectively, with a vaporizer temperature of 250 °C of the heated ESI source. The ion transfer capillary temperature was 250 °C. The S‐lens RF was set at 55 (arbitrary unit). The orbitrap resolution was *m*/Δ*m* 140000. The Automatic Gain Control target was 5 ⋅ 10^6^ and the maximum injection time was set to 0.1 s. CID experiments were performed using collision energies in the laboratory frame of 50 and 70 eV and 0.01 s activation time. For monoisotopic operation, a window of *m/z* 1.0 was applied for precursor isolation. The CID mass spectra are normalized to the respective TIY which is obtained by summing up the ion yields in the *m/z* range 49.5 to 615.

## Conflict of interest

The authors declare no conflict of interest.

## Supporting information

As a service to our authors and readers, this journal provides supporting information supplied by the authors. Such materials are peer reviewed and may be re‐organized for online delivery, but are not copy‐edited or typeset. Technical support issues arising from supporting information (other than missing files) should be addressed to the authors.

Supporting Information
