# Supplementary material for: Ionization and Photofragmentation of Isolated Metalloporphyrin Cations Investigated by VUV Action Spectroscopy
Source: Chemistry. 2021 Jul 14;27(48):12371–9. doi: 10.1002/chem.202101515 (PMC8457234; doi:10.1002/chem.202101515)
Supplement: Supplementary file 1 — Supporting Information [file CHEM-27-12371-s001.pdf]

Chemistry–A European Journal

Supporting Information

Ionization and Photofragmentation of Isolated Metalloporphyrin Cations Investigated by VUV Action Spectroscopy**

Kaja Schubert[+], Lucas Schwob[+],* Simon Dörner, Marion Girod, Luke MacAleese, Cornelius L. Pieterse, Thomas Schlathölter, Simone Techert, and Sadia Bari*

Table of content

- Figure S1: Full range mass spectra of $FePPIX^+$
- Figure S2: theoretical and experimental isotopic patterns of $Fe^{III}PPIX^+$
- High harmonic photon contributions: correction procedure
- Table S1: fragment assignment from the orbitrap Q-Exactive® data
- Figure S3: PIY spectra of $FePPIX^+$, $CoPPIX^+$ and $ZnPPIX^+$ singly charged photo products
- Figure S4: PIY spectra of $FePPIX^+$, $CoPPIX^+$ and $ZnPPIX^+$ doubly charged photo products
- Figure S5: Precursor ion peaks from ESI-only mass spectra of $FePPIX^+$, $CoPPIX^+$ and $ZnPPIX^+$
- Figure S6: Mass spectra of $FePPIX^+$, $CoPPIX^+$ and $ZnPPIX^+$ at 34.9 eV.

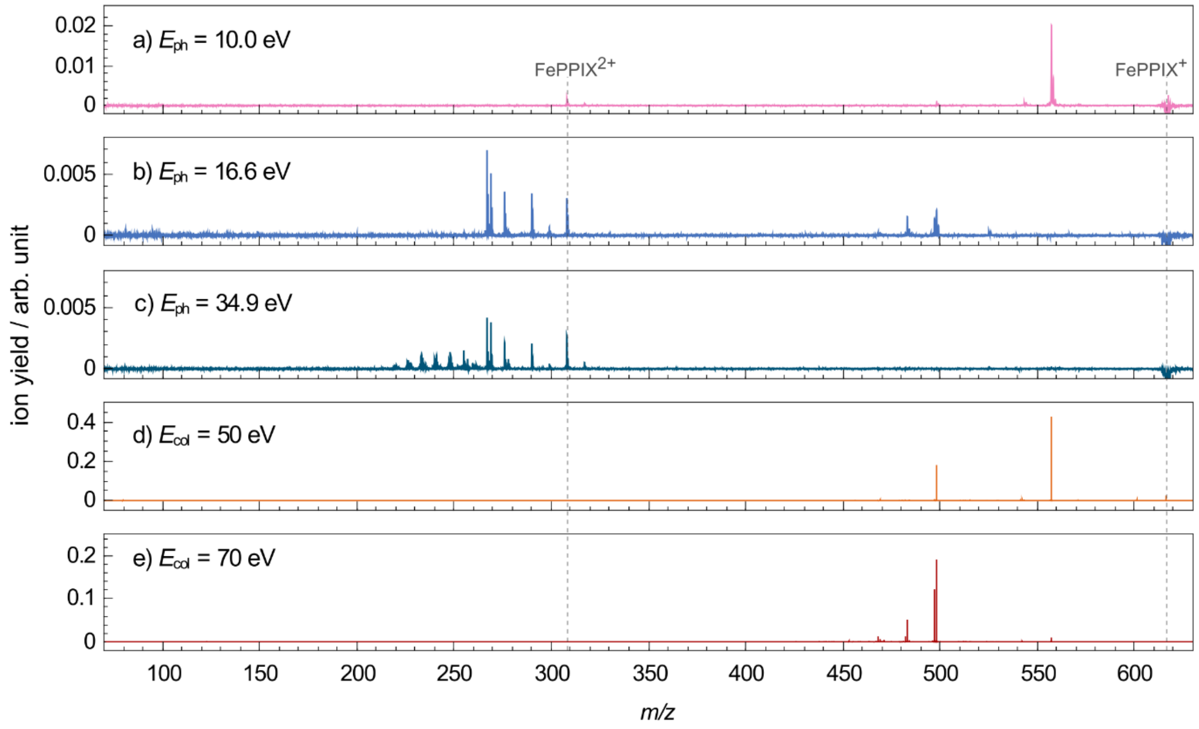

Figure S1. Full range mass spectra after irradiation of $FePPIX^+$ at a) 10.0 eV b) 16.6 eV and c) 34.9 eV and after collisional activation at d) 50 eV and e) 70 eV. The precursor ion peak at *m/z* 616 appears negative in a)-c) because of subtraction of mass spectrum without irradiation (ESI only).

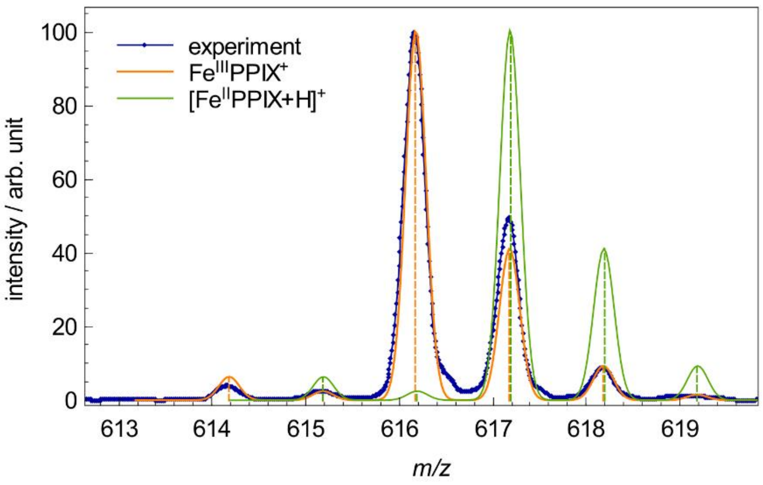

Figure S2. Theoretical isotopic patterns of $Fe^{III}PPIX^+$ (orange line) and $[Fe^{II}PPIX + H]^+$ (green line) in comparison with a measured, *m/z* filtered mass spectrum of electrosprayed iron protoporphyrin IX (blue diamonds). The isotopic patterns are broadened with our experimental resolution of $m/\Delta m$ ~ 2400 with a Gaussian function. The deviations in the isotopic ratios between the theoretical and experimental pattern of $Fe^{III}PPIX^+$ are due to the quadrupole mass filter settings.

High harmonic contributions

At photon energies between 11 and 15 eV high harmonic photons (mainly from the second harmonic) contribute to the photon beam. The partial ion yields were corrected regarding these contributions in the following way. As an example, correction of the ion yields at a photon energy of 12 eV is shown here. Note that this procedure can only be applied in the present study as in this energy range, fragments which are exclusively produced by the high harmonic photons were observed.

The measured ion yield of a photo product $Y_{12\mathrm{eV}}(m/z)$ is given by the yield from absorption of the low-energy photons $Y^{*}_{12\mathrm{eV}}(m/z)$ and by the yield from absorption of the high harmonic photons $Y^{hh}_{12\mathrm{eV}}(m/z)$.

$$Y_{12\mathrm{eV}}(m/z) = Y^{*}_{12\mathrm{eV}}(m/z) + Y^{hh}_{12\mathrm{eV}}(m/z) \qquad (1)$$

For most doubly charged fragments it applies that the number of bond cleavages increases with increasing excitation energy (see main text and Figure 5 in the main text). Therefore, we conclude that photo fragments at *m/z* < 265 at photon energies between 11 and 15 eV are predominantly formed by absorption of the high harmonic photons. Accordingly, $Y^{hh}_{12\mathrm{eV}}(m/z)$ in equation (1) can be determined by using the branching ratio of the yield of such a fragment (e.g. m/z 255) at the low and high harmonic photon energy $Y^{hh}_{12\mathrm{eV}}(m/z\ 255) : Y_{24\mathrm{eV}}(m/z\ 255)$ and by using the measured yield of a photofragment in the high-energy spectrum $Y_{24\mathrm{eV}}$.

$$Y^{hh}_{12eV}(m/z) = Y_{24\mathrm{eV}}(m/z) \cdot \frac{Y^{hh}_{12\mathrm{eV}}(m/z\ 255)}{Y_{24\mathrm{eV}}(m/z\ 255)} \qquad (2)$$

The real contribution of the low-energy fragments in the energy range of 11 to 15 eV is then given by:

$$Y^{*}_{12\mathrm{eV}}(m/z) = Y_{12\mathrm{eV}}(m/z) - Y_{24\mathrm{eV}}(m/z) \cdot \frac{Y^{hh}_{12\mathrm{eV}}(m/z\ 255)}{Y_{24\mathrm{eV}}(m/z\ 255)} \qquad (3)$$

The ion yields at photon energies between 11 and 15 eV were corrected accordingly.

Table S1 Fragment *m/z* and assignments upon collisional activation of $FePPIX^+$ for collision energies E_{col} of 50 eV and 70 eV. Assignments marked with * can have a different assignment with the same theoretical *m/z* (e.g. *m/z* 497.1428 ≙ -2cCa-H or -cCa-Ca-m). However, at collision energies of 100 eV (not shown here) a peak at *m/z* 437.0446 corresponding to four methyl losses from the *m/z* 497 peak is observed. Since only four methyl side chains are available, we assigned the *m/z* 497 peak to the -2ccCa-H and not the -cCa-Ca-m loss. The same argument applies for other assignments involving hydrogen loss(es).

Experimental *m/z*	Theoretical *m/z*	Losses from the precursor ion	mass difference (ppm)
**E_{col} = 50 eV**			
557.1571	557.1640	-cCa	-12.5
543.1422	543.1484	-ccCa	-11.4
498.1446	498.1507	-2cCa	-12.3
497.1372	497.1428	-2cCa-H*	-11.3
484.1293	484.1350	-cCa-ccCa	-11.8
**E_{col} = 70 eV**			
557.1575	557.1640	-cCa	-11.6
498.1443	498.1507	-2cCa	-12.8
497.1377	497.1428	-2cCa-H*	-10.2
496.1302	496.1350	-2cCa-2H*	-9.7
484.1297	484.1350	-cCa-ccCa	-11.0
483.1219	483.1272	-2cCa-m	-10.9
482.1143	482.1194	-2cCa-H-m*	-10.7
481.1065	481.1115	-2cCa-2H-m*	-10.4
471.1220	471.1272	-2cCa-v	-11.1
470.1142	470.1194	-2ccCa	-11.1
469.1063	469.1115	-m-cCa-ccCa	-11.0
468.0985	468.1037	-2cCa-2m	-11.1
467.0907	467.0959	-2cCa-H-2m*	-11.1

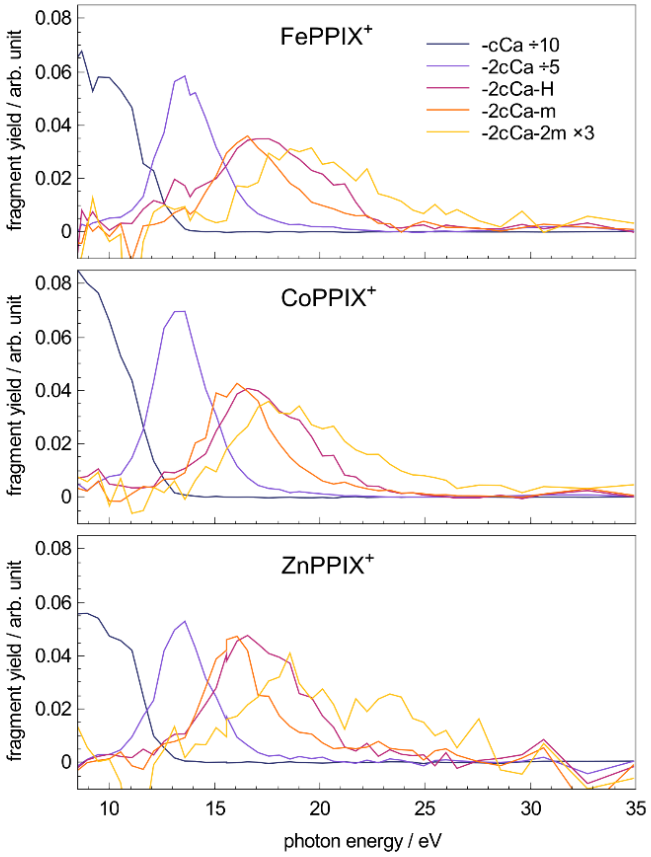

Figure S3. PIY spectra for $FePPIX^+$ (top), $CoPPIX^+$ (middle), $ZnPPIX^+$ (bottom) of the singly charged photo products upon VUV photoabsorption.

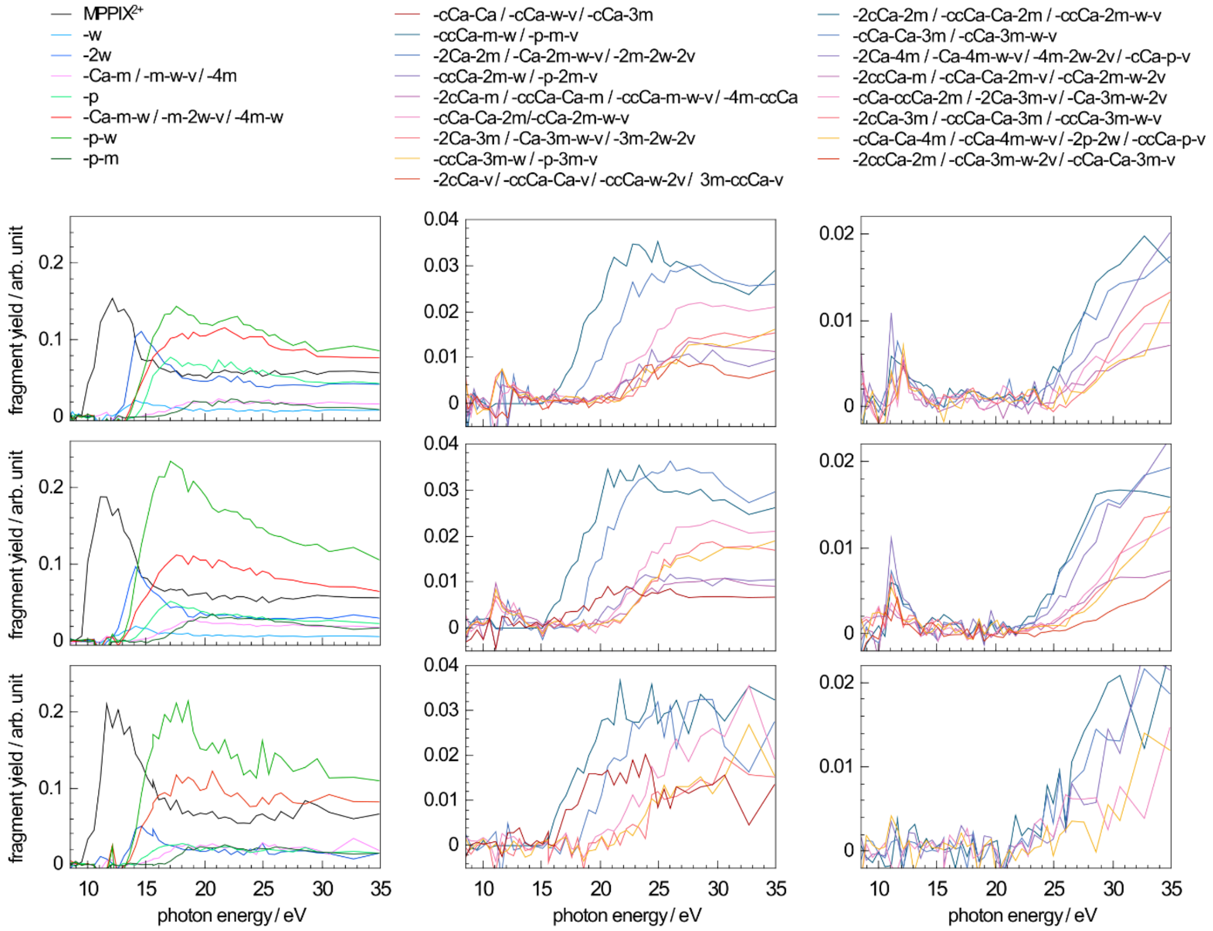

Figure S4. PIY spectra for FePPIX+ (top), CoPPIX+ (middle), ZnPPIX+ (bottom) of the doubly charged photo products upon VUV photoabsorption. Due to their low intensity the yields of the low *m/z* fragments (*m/z* < 242) are more sensitive towards statistical fluctuations. Therefore, the correction for high harmonic photon contributions does not work properly for these fragments at 10-15eV. We assume that the contributions in this energy region are attributed to the absorption of high harmonic photons.

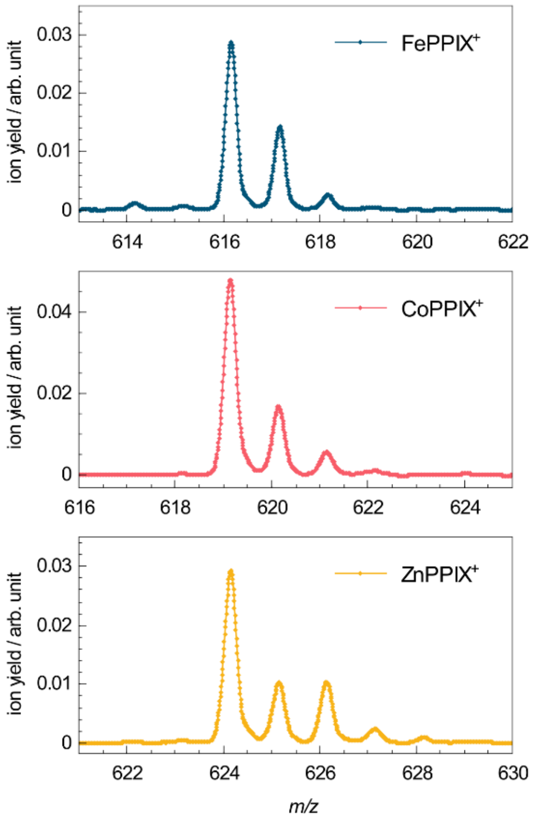

Figure S5. Precursor ion peaks from ESI-only mass spectra of $FePPIX^+$, $CoPPIX^+$ and $ZnPPIX^+$.

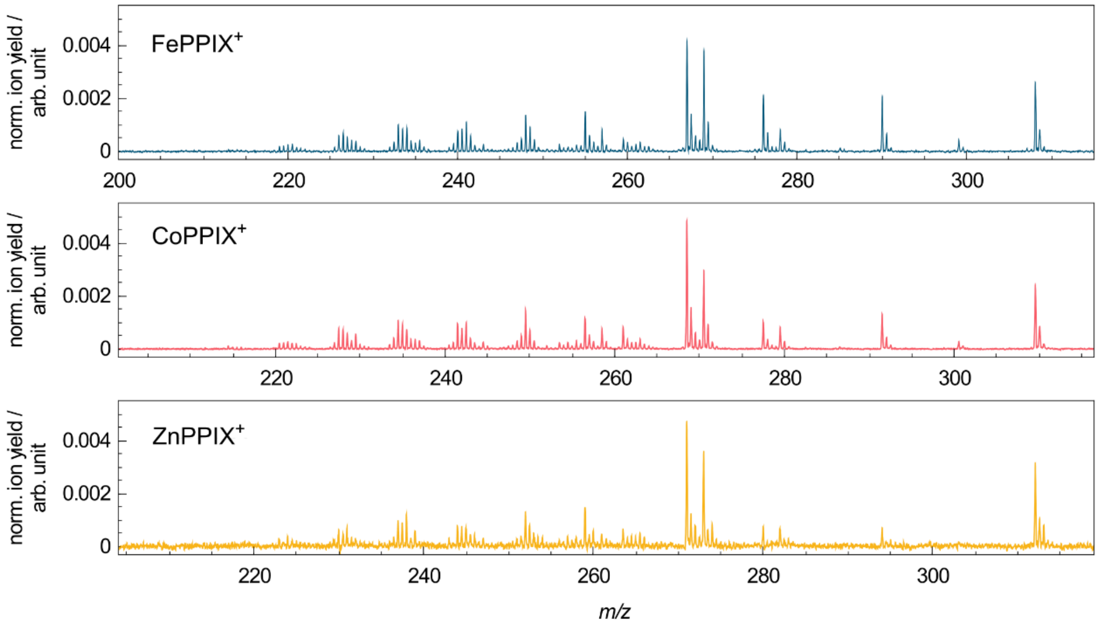

Figure S6. Mass spectra for $FePPIX^+$, $CoPPIX^+$ and $ZnPPIX^+$ upon irradiation with 34.9 eV photons, zoomed in the doubly charged photo products region. For better comparison, the *m/z* axes are shifted by half of the difference in mass between Co and Fe (Δm = 3.0 u) and Zn and Fe (Δm = 8.0 u), respectively.
